# Safety and tolerability of cryocompression as a method of enhanced limb hypothermia to reduce taxane-induced peripheral neuropathy

**DOI:** 10.1007/s00520-019-05177-2

**Published:** 2019-12-06

**Authors:** Aishwarya Bandla, Stacey Tan, Nesaretnam Barr Kumarakulasinghe, Yiqing Huang, Sally Ang, Gayathiri Magarajah, Zarinah Hairom, Joline Si Jing Lim, Alvin Wong, Gloria Chan, Natalie Ngoi, Emily Ang, Yee Mei Lee, Amanda Chan, Soo-Chin Lee, Nitish Thakor, Einar Wilder-Smith, Raghav Sundar

**Affiliations:** 1 The N.1 Institute for Health, National University of Singapore, Singapore, Singapore; 2 Department of Haematology-Oncology, National University Health System, Singapore, Singapore; 3 National University Cancer Institute, National University Health System, Singapore, Singapore; 4 Department of Medicine, National University Health System, Singapore, Singapore; 5 Cancer Science Institute of Singapore, National University of Singapore, Singapore, Singapore; 6 Department of Biomedical Engineering, Johns Hopkins University, Baltimore, USA; 7 Neurology, Kantonsspital, Lucerne, Switzerland; 8 Yong Loo Lin School of Medicine, National University of Singapore, Singapore, Singapore

**Keywords:** Chemotherapy-induced peripheral neuropathy, Paclitaxel, Cryotherapy, Cryocompression, Nerve conduction

## Abstract

**Purpose:**

Severe peripheral neuropathy is a common dose-limiting toxicity of taxane chemotherapy, with no effective treatment. Frozen gloves have shown to reduce the severity of neuropathy in several studies but comes with the incidence of undesired side effects such as cold intolerance and frostbite in extreme cases. A device with thermoregulatory features which can safely deliver tolerable amounts of cooling while ensuring efficacy is required to overcome the deficiencies of frozen gloves. The role of continuous-flow cooling in prevention of neurotoxicity caused by paclitaxel has been previously described. This study hypothesized that cryocompression (addition of dynamic pressure to cooling) may allow for delivery of lower temperatures with similar tolerance and potentially improve efficacy.

**Method:**

A proof-of-concept study was conducted in cancer patients receiving taxane chemotherapy. Each subject underwent four-limb cryocompression with each chemotherapy infusion (three hours) for a maximum of 12 cycles. Cryocompression was administered at 16 °C and cyclic pressure (5–15 mmHg). Skin surface temperature and tolerance scores were recorded. Neuropathy was assessed using clinician-graded peripheral sensory neuropathy scores, total neuropathy score (TNS) and nerve conduction studies (NCS) conducted before (NCS_pre_), after completion (NCS_post_) and 3 months post-chemotherapy (NCS_3m_). Results were retrospectively compared with patients who underwent paclitaxel chemotherapy along with continuous-flow cooling and controls with no hypothermia.

**Results:**

In total, 13 patients underwent 142 cycles of cryocompression concomitant with chemotherapy. Limb hypothermia was well tolerated, and only 1 out of 13 patients required an intra-cycle temperature increase, with no early termination of cryocompression in any subject. Mean skin temperature reduction of 3.8 ± 1.7 °C was achieved. Cryocompression demonstrated significantly greater skin temperature reductions compared to continuous-flow cooling and control (*p* < 0.0001). None of the patients experienced severe neuropathy (clinician-assessed neuropathy scores of grade 2 or higher). NCS analysis showed preservation of motor amplitudes at NCS_3m_ in subjects who underwent cryocompression, compared to the controls who showed significant deterioration (NCS_3m_ cryocompression vs. NCS_3m_ control: ankle stimulation: 8.1 ± 21.4%, *p* = 0.004; below fibula head stimulation: 12.7 ± 25.6%, *p* = 0.0008; above fibula head stimulation: 9.4 ± 24.3%, *p* = 0.002). Cryocompression did not significantly affect taxane-induced changes in sensory nerve amplitudes.

**Conclusion:**

When compared to continuous-flow cooling, cryocompression permitted delivery of lower temperatures with similar tolerability. The lower skin surface temperatures achieved potentially lead to improved efficacy in neurotoxicity amelioration. Larger studies investigating cryocompression are required to validate these findings.

**Electronic supplementary material:**

The online version of this article (10.1007/s00520-019-05177-2) contains supplementary material, which is available to authorized users.

## Introduction

New research shows a growing interest towards cryotherapy or regional hypothermia for alleviating chemotherapy-induced toxicities such as oral mucositis, ocular toxicity, onycholysis and peripheral neuropathy [11]. Of these debilitating side effects, chemotherapy-induced peripheral neuropathy (CIPN) is a common dose-limiting toxicity of several neurotoxic chemotherapeutic agents, manifesting as severe pain and tingling sensation in the finger and toe tips [30]. A recent meta-analysis of more than 4000 chemotherapy-treated patients found the prevalence of CIPN to be 68.1% within the first month of chemotherapy treatment, 60.0% at 3 months and 30.0% at 6 months [24]. Currently, with no available cure, the treatment is limited to symptomatic pharmacological interventions and dose reduction which in turn reduces the efficacy of the chemotherapy itself.

In a recent trial reported by Hanai et al. (2018), cryotherapy in the form of frozen gloves was effective in preventing and reducing the occurrence of CIPN [9]. Similarly, inspired by scalp cooling therapy to combat chemotherapy-induced alopecia (CIA) [7, 15], several groups have demonstrated promising results that the use of frozen gloves and socks concomitantly with the chemotherapy infusion is efficacious in reducing CIPN [4, 9, 23, 29, 31, 32]. However, latest reports indicate that the frozen gloves have been recalled due to incidences of frostbite and other patient safety issues [10]. Moreover, this form of cooling has other major limitations such as lack of stable thermoregulation (ability to change the temperature of the hypothermia delivered according to tolerability) and the need to frequently replace the frozen gloves or ice packs, leading to “breaks” in the hypothermia delivery [3].

The role of continuous-flow limb cooling in decreasing the incidence of CIPN has been previously described in studies by our group [2, 25]. Coolant temperature of 22 °C was determined to be the lowest tolerable temperature in healthy volunteers for a duration of 3 h (corresponding to the duration of chemotherapy) [2]. Using these data, a trial was conducted in cancer patients undergoing adjuvant paclitaxel chemotherapy with concomitant continuous-flow cooling purely on the lower limbs [25]. To have an early initial signal of efficacy, an internal randomization was performed by administering unilateral lower limb cooling while the other was a paired-control. Only a moderate degree of cooling was achieved, with continuous-flow cooling alone, due to tolerability issues with lower temperatures. However, cancer subjects showed mild improvement in nerve conduction in the cooled leg compared to the control [25]. After much internal research, it was discovered that the addition of pressure to cooling (cryocompression) allowed for a greater level of tolerability and degree of cooling. Here, the results of our study exploring cryocompression as a means to achieve greater magnitude of regional hypothermia with similar tolerance levels towards efficacious alleviation of CIPN is described. Cryocompression of the limbs was carried out concomitantly during chemotherapy. Tolerability and skin temperature changes were assessed, accompanied by neurophysiological and clinical assessments of neuropathy. The results of the above-mentioned assessments in subjects who underwent cryocompression were also compared with those who underwent continuous-flow cooling during chemotherapy and controls (from our previous study [25]).

## Material and method

### Study design

The study was conducted at the National University Hospital, Singapore from November 2015 to February 2017 in accordance with the protocol approved by the Institutional Review Board of the National Health Group, Singapore. The procedures followed were in accordance with the ethical standards of the responsible committee on human experimentation and the 1964 Helsinki declaration and its later amendments. Cancer patients scheduled to receive taxane-based (weekly paclitaxel, 80 mg/m^3^ or 3-weekly docetaxel, 75 mg/m^3^) chemotherapy were invited to participate in the study. All subjects were informed orally and in writing about the aims of the study and written consent for participation was sought and documented.

The inclusion/exclusion criteria were designed as follows. The study population comprised of cancer patients who were eligible for recruitment if they fulfilled the following criteria: (i) aged 21–80 years; (ii) signed informed consent from patient or legal representative; (iii) no history of neuropathy; (iv) Eastern Cooperative Oncology Group (ECOG) performance status 0; and (v) no history of hospitalization in the past 6 months. Patients were ineligible for the study if they had: (i) open skin wounds or ulcers of the limbs; or (ii) history of Raynaud’s phenomenon, peripheral vascular disease, or poorly-controlled diabetes (HbA1c > 10%).

**Fig. 1:**
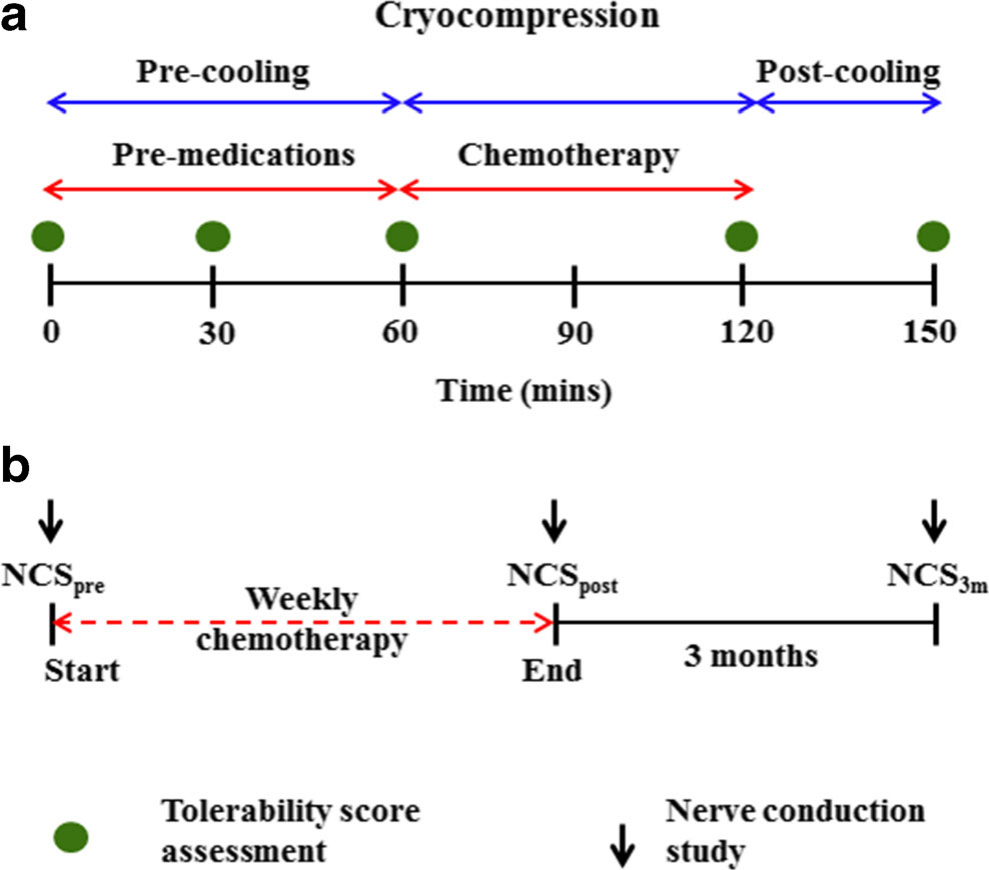
Trial flow diagram. **a** Schematic of a single chemo-cryocompression session indicating that limb hypothermia was administered as three parts: (i) pre-cooling (60 min) along with administration of pre-medication drugs, followed by (ii) cooling during taxane infusion (60 min) and finally a (iii) post-cooling phase (30 min). The green dots indicate the time points at which tolerability scores were assessed. **b** Overall schematic of the study which constituted up to 12 weeks of chemotherapy. NCS assessments were conducted before the start (NCS_pre_), at the end of chemotherapy (NCS_post_) and after three months (NCS_3m_)

During every cycle of chemotherapy, premedication drugs (dexamethasone, diphenhydramine and ranitidine) were administered 30 min prior to the taxane infusion. The taxane chemotherapy was administered as a 1-h infusion (Fig. 1). The chemotherapy unit’s ambient temperature was adjusted to 21 °C via air-conditioning. Enrolled cancer patients received four-limb cryocompression at 16 °C and cyclic pressure (5–15 mmHg) (these parameters were determined as the lowest tolerable in a healthy subject study) concurrently with their chemotherapy. The study protocol in Fig. 1 indicates the sequence of treatments, comprising of a pre-cooling period (1 h), continued with the taxane infusion and a post-cooling period (on average, 30 min after the end of the taxane infusion). Overall, hypothermia was administered for no longer than 4 h. Safety and tolerability of the cryocompression in cancer patients were assessed during the cryocompression regime as shown in Fig. 1, through well-defined tolerability measures including three validated scales: visual analogue pain scale (VAS), subjective tolerance scale (STS) and the shivering assessment scale (SAS) [25]. A detailed safety protocol was followed for coolant thermoregulation, should the patient find the hypothermia intolerable [25].

Cryocompression was applied to the subjects via a Game Ready 2.0 Refurbished System (CoolSystems, Inc., CA, USA), which simultaneously applied active cyclical pneumatic compression (cycling from 5–15 mmHg every 5 min) and cooling to the limbs through limb wraps attached to a thermoregulator. The system comprised of a portable thermoregulator control unit together with arm and leg cooling wraps, geometrically designed to comfortably fit around the subject’s limbs and could be easily applied and taken off. The control unit circulated the coolant (water) and air through the cooling wraps to cool and compress the wrapped limb. Skin surface temperature was measured using wireless temperature sensors (VitalSense, Philips Respironics, US) placed at evenly spaced locations on all four limbs [2, 25]. Temperature was continuously monitored throughout the duration of cryocompression and the data recorded through a wireless monitor every minute. Core body temperature (aural) was monitored and recorded once every hour throughout cryocompression.

### Assessment of neuropathy

Nerve conduction study (NCS), including evaluation of sensory and motor nerve functions, was conducted at three time points—before the start of chemotherapy (NCS_pre_), after chemotherapy (NCS_post_) and 3 months after chemotherapy (NCS_3m_) (Fig. 1). Tests were performed as previously standardized by Karandreas et al. (1995) and Ping Ng et al. (2013); sensory nerve action potential (SNAP) amplitudes and sensory nerve conduction velocities were measured in the bilateral sural, superficial peroneal, saphenous, medial and lateral plantar nerves [12]. Compound motor action potential (cMAP) amplitudes and motor nerve conduction velocities were evaluated in the bilateral common peroneal and tibial nerves [22]. To allow for homogeneity and comparability of study populations, we only focused on patients who underwent weekly-paclitaxel chemotherapy for our exploratory efficacy analysis.

We also compared the efficacy of cryocompression and continuous-flow cooling by comparing data from this study to our previous study where cancer patients received unilateral leg cooling (without compression) together with their chemotherapy while the study’s uncooled limbs were treated as the control group [25].

Clinical evaluation using the modified total neuropathy score (TNS) was performed at the same time points as the NCS [28]. Clinician-assessed peripheral sensory neuropathy was recorded once every 3 weeks using Common Terminology Criteria for Adverse Events (CTCAE) version 4.03.

### Statistical analysis

The temporal trend of skin temperature changes, over the duration of hypothermia was summarized as a mean of the recorded temperatures for all cycles of hypothermia for all patients. Similarly, tolerability to limb hypothermia was analysed as an average of all patients’ tolerance scores across all cycles of hypothermia. Sensory and motor nerve parameters of amplitude and velocity, at every NCS visit, were analysed as relative percentage changes with respect to the first NCS visit (NCS_pre_) and averaged across patients.

Continuous variables are shown as mean ± S.D. (standard deviation). A paired *t* test was used to compare the temperature and NCS values between time points of each individual subject. NCS_3m_ values of subjects who underwent cryocompression were compared with those of control group using Welch’s *t* test. A two-tailed *p* value < 0.05 was considered statistically significant. All statistical analyses were performed in Microsoft Excel (V.12.0 for Windows, Microsoft Corp., Washington, USA).

## Results

Thirteen cancer patients (mean age 55 years, range 33–69 years) scheduled to receive taxane-based chemotherapy participated in the study. The patient characteristics are detailed in Table 1. All patients completed their scheduled cycles of chemotherapy and nerve conduction assessments without early termination or withdrawal. On an average, the patients received a cumulative taxane dose of 856.0 (375.0–960.0) mg/m^2^ of either paclitaxel or docetaxel.

**Table 1:** Baseline patient characteristics

Variables	*N* (%) (total *N* = 13)	Mean (range)
Age (years)	–	55 (33–69)
Weight (kg)	–	70 (64–78)
Height (cm)	–	158 (148–168)
BSA (m^2^) baseline	–	1.75 (1.65–1.91)
Cumulative dose of taxane (mg/m^2^)	–	856.0 (375.0–960.0)
Cancer type/chemo regimen		–
Breast/weekly paclitaxel	11 (85)	
Prostate/3-weekly docetaxel	2 (15)	
TNS baseline		–
0	7 (54)	
1	1 (8)	
2	3 (23)	
3	2 (15)	

### Safety and tolerability

Cryocompression (at 16 °C and dynamic compression) was overall well tolerated as measured by a well-defined range of tolerability measures (Supplementary Figure S1 a–c). Premature termination of cooling was not necessary for any of the patients. Only one patient (for 1 out of a total 142 cycles) required an intra-cycle thermoregulator temperature increase of 1 °C towards the end of a hypothermia session. Temporary erythema lasting a few minutes was observed upon removal of the cooling wraps. Overall, no serious or lasting adverse events were encountered as a result of limb hypothermia (Supplementary Table S1). On average, patients’ core body temperature recorded negligible changes of 0.07 ± 0.41 °C averaged across all chemotherapy cycles. In comparison, subjects who underwent continuous-flow cooling displayed mild intolerance to 22 °C (large error bars in Supplementary Figure S1 f). Continuous-flow cooling temperatures lower than 22 °C was not tolerated by healthy volunteers in our previous study [2].

### Skin temperature changes with limb hypothermia

**Fig. 2:**
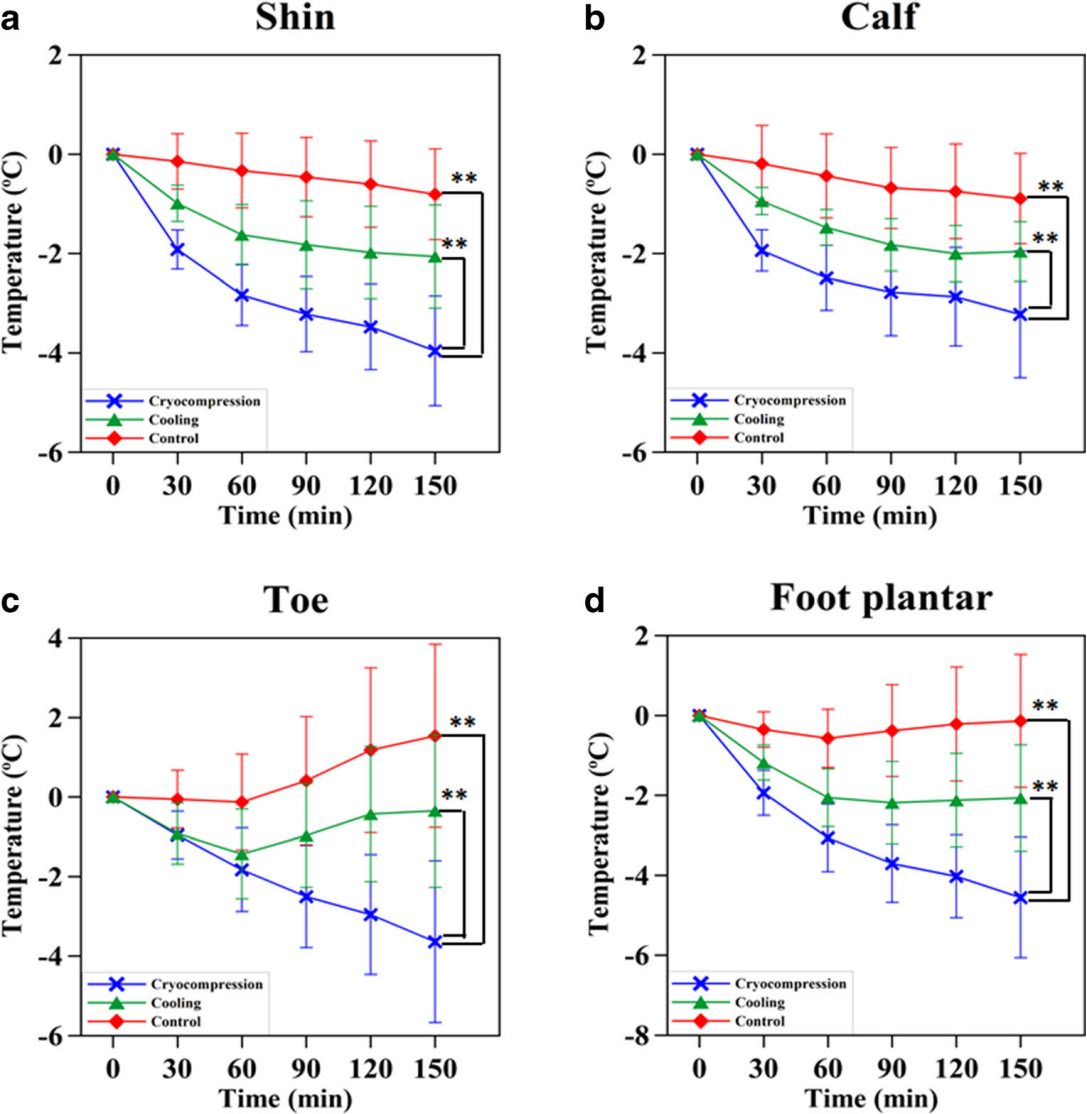
Comparison of skin temperature changes with and without limb hypothermia via cooling and cryocompression techniques. The relative changes in skin surface temperature in the **a**) shin, **b**) calf, **c**) toe and **d**) foot plantar regions over the duration of chemotherapy indicate that cryocompression (blue) offers the best temperature drop compared to continuous-flow cooling (green). The non-cooled limb was considered as control (red) which showed a minor temperature drop as well, owing to the low room temperature. ^**^ indicates *p* < 0.0001

Skin temperature changes on the limbs were recorded and averaged across all patients over all 142 cycles. At the end of duration of cryocompression, a mean skin temperature drop of 2.9 ± 2.3 °C and 3.8 ± 1.7 °C was achieved on the arms and legs, respectively. On an average, cryocompression achieved a skin temperature decrease of 2.32 °C lower than continuous-flow cooling (Fig. 2). Overall, cryocompression demonstrated significantly greater skin temperature reductions compared to both the continuous-flow cooling and the control groups (*p* < 0.0001)

### Clinical neuropathy

The TNS grade of CIPN reported during all the three visits were documented (Supplementary Figure S2). Baseline TNS ranged between zero and three for all patients, indicating absence of any baseline neuropathy. Clinician-investigator assessed CTCAE measurement of sensory CIPN was also collected [6]. None of the patients experienced grade 2 or higher sensory CIPN as the worst-recorded grade. Six (46%) had grade 0, and 7 (54%) had CTCAE grade 1 CIPN (Supplementary Table S1).

### Nerve conduction changes

**Fig. 3:**
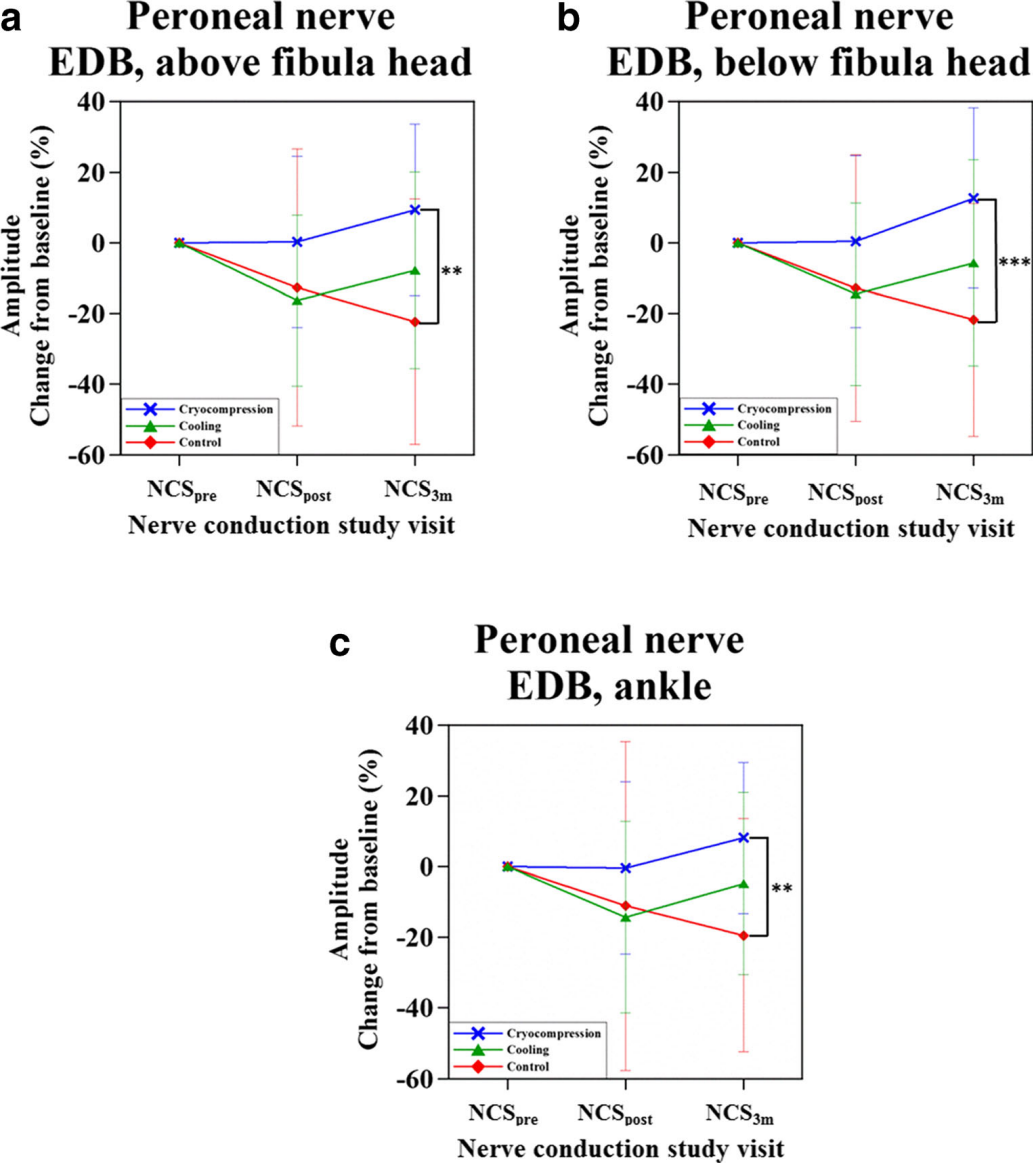
Comparison of nerve conduction changes with and without limb hypothermia via cooling and cryocompression techniques. The changes in nerve conduction amplitudes in the motor nerves (**a**–**c**) at three time points—before, end of chemotherapy and after three months indicate that cryocompression (blue) better preserves motor nerve conduction amplitudes compared to continuous-flow cooling (green). The non-cooled limb was considered as control (red), continued to deteriorate. ^**^ indicates *p* < 0.01 and ^***^ indicates *p* < 0.001

NCS analysis was performed for 11 (out of 13) subjects who received paclitaxel chemotherapy (two subjects underwent docetaxel chemotherapy and were included for safety analysis only). Changes at NCS_post_ and NCS_3m_ were calculated as relative changes from baseline (NCS_pre_). NCS analysis following cryocompression showed preservation of cMAP amplitudes at NCS_post_ and NCS_3m_ (NCS_post_ vs. NCS_pre_ and NCS_3m_ vs. NCS_pre_ of the peroneal nerve were not significant; *p* > 0.05, indicating preservation of NCS parameters) (Fig. 3 (blue), Supplementary Figure S3 and Table 2). On the other hand, the control group showed significant decrease in cMAPs at NCS_3m_ and NCS_post_ compared to NCS_pre_ (Fig. 3 (red), Supplementary Figure S3 and Table 2). Moreover, the cMAPs at NCS_3m_ of subjects who underwent cryocompression was significantly higher compared to the controls (NCS_3m_ cryocompression vs. NCS_3m_ control: ankle stimulation: 8.1 ± 21.4%, *p* = 0.004; below fibula head stimulation: 12.7 ± 25.6%, *p* = 0.0008; above fibula head stimulation: 9.4 ± 24.3%, *p* = 0.002) (Fig. 3, Supplementary Figure S3 and Table 2). SNAP amplitudes in all subjects continued to significantly deteriorate at NCS_post_ and NCS_3m_ compared to NCS_pre_ in all three groups (i.e., control, cooling and cryocompression). Limb hypothermia did not seem to significantly change outcomes compared to the control group (without hypothermia) (Table 3). Motor and sensory nerve velocities remained largely unaffected at NCS_post_ and NCS_3m_ in all three groups. Further, cryocompression did not affect nerve velocities at NCS_3m_ (Tables 2–3).

**Table 2:** Absolute changes (from baseline NCS, NCS_pre_) in motor nerve conduction amplitude and velocity at the end of chemotherapy (NCS_post_) and after three months (NCS_3m_). Percentage changes are shown in brackets

Motor amplitude	δ (3 months)			δ (6 months)		
	Cryocompression μV (%)	Cooling μV (%)	Control μV (%)	Cryocompression μV (%)	Cooling μV (%)	Control μV (%)
EDB (ankle)	0.07 (− 0.43)	− 0.81 (− 14.28)	− 0.66 (− 11.07)	0.30 (8.14)	− 0.43 (− 4.79)	− 0.83 (− 19.48)
EDB (below fib head)	0.09 (0.37)	− 0.73 (− 14.47)	− 0.61 (− 12.8)	0.37 (12.71)	− 0.46 (− 5.7)	− 0.85 (− 21.8)
EDB (above fib head)	0.08 (0.31)	− 0.77 (− 16.3)	− 0.62 (− 12.67)	0.25 (9.37)	− 0.49 (− 7.79)	− 0.86 (− 22.3)
AH (ankle)	− 1.2 (− 9.45)	− 1.07 (− 8.38)	− 1.76 (− 12.81)	− 1.69 (− 8.76)	− 1.12 (− 10.40)	− 1.96 (− 14.09)
AH (knee)	− 0.67 (− 3.02)	− 0.22 (− 2.07)	− 0.92 (− 7.73)	− 1.40 (− 12.57)	− 0.58 (− 6.17)	− 1.83 (− 16.53)
Motor velocity	Cryocompression m/s (%)	Cooling m/s (%)	Control m/s (%)	Cryocompression m/s (%)	Cooling m/s (%)	Control m/s (%)
EDB (below fib head)	− 1.32 (− 2.82)	− 1.43 (− 2.85)	− 0.97 (− 1.93)	1.81 (4.66)	0.22 (0.9)	0.14 (0.5)
EDB (above fib head)	− 0.95 (− 0.58)	− 3.28 (− 5.84)	− 0.08 (0.67)	− 1.54 (− 1.31)	− 1.99 (− 2.43)	2.56 (6.6)
AH (knee)	− 0.99 (− 1.23)	− 2.8 (− 5.75)	− 1.46 (− 2.81)	0.13 (1.61)	− 0.66 (− 1.13)	− 0.61 (− 1.1)

**Table 3:** Absolute changes (from baseline NCS, NCS_pre_) in sensory nerve conduction amplitude and velocity at the end of chemotherapy (NCS_post_) and after 3 months (NCS_3m_). Percentage changes are shown in brackets

Sensory amplitude	δ (3 months)		δ (6 months)			
	Cryocompression μV (%)	Cooling μV (%)	Control μV (%)	Cryocompression μV (%)	Cooling μV (%)	Control μV (%)
Sural	− 5.68 (− 28.09)	− 3.92 (− 19.20)	− 4.39 (− 22.9)	− 5.13 (− 26.69)	− 4.15 (− 22.66)	− 5.43 (− 28.5)
Superficial peroneal	− 5.34 (− 23.29)	− 7.61 (− 32.14)	− 7.68 (− 32.9)	− 7.87 (− 28.15)	− 9.5 (− 39.21)	− 8.78 (− 37.57)
Saphenous	− 1.15 (− 21.95)	− 1.38 (− 20.08)	− 1.73 (− 29.65)	− 1.62 (− 27.49)	− 1.77 (− 27.3)	− 1.86 (− 27.58)
Medial plantar	− 4.41 (− 34.63)	− 6.95 (− 54.78)	− 7.26 (− 55.78)	− 5.04 (− 44.81)	− 7.82 (− 58.95)	− 7.98 (− 59.35)
Lateral plantar	− 2.97 (− 16.83)	− 2.02 (− 37.82)	− 2.35 (− 41.88)	− 3.28 (− 48.14)	− 2.36 (− 51.57)	− 2.54 (− 53.91)
Sensory velocity	Cryocompression m/s (%)	Cooling m/s (%)	Control m/s (%)	Cryocompression m/s (%)	Cooling m/s (%)	Control m/s (%)
Sural	− 3.88 (− 4.84)	1.99 (1.89)	− 0.69 (− 0.66)	0.81 (3.63)	6.26 (8.62)	3.83 (8.61)
Superficial peroneal	− 4.76 (− 5.37)	0.12 (0.95)	− 1.07 (− 1.43)	− 1.29 (0.3)	4.5 (8.95)	4.77 (8.59)
Saphenous	− 2.58 (− 2.78)	− 0.09 (1.86)	1.36 (5.85)	1.92 (6.44)	5.05 (12.02)	8.33 (21.12)
Medial plantar	2.15 (4.99)	− 7.92 (− 6.89)	− 5.46 (− 9.49)	− 6.28 (− 8.27)	− 7.11 (− 5.15)	− 2.74 (− 4.47)
Lateral plantar	− 3.06 (− 3.93)	− 2.73 (− 4.51)	0.09 (0.85)	− 4.7 (− 4.82)	− 2.15 (− 3.24)	0.08 (0.51)

Changes in motor amplitudes (over the EDB shown in Fig. 3) at NCS_3m_ showed a correlation with skin temperature changes (*r* = 0.4).

## Discussions

In this study, we have demonstrated that the addition of pressure to cooling (cryocompression) allows for delivery of significantly lower degrees of hypothermia, resulting in lower skin temperatures. This mode of delivery of regional hypothermia is well tolerated and can be administered over multiple cycles of chemotherapy. Moreover, cryocompression may potentially improve the efficacy of preventing CIPN compared to continuous-flow limb cooling, however, this remains to be demonstrated in further studies.

Studies describe compression contributing to improved tolerability of pain over long durations of hypothermia administered post-surgery by reducing the formation of oedema [5]. In particular, a study by Murgier and Cassier (2014) showed that dynamic compression together with cooling was more effective in reducing postoperative pain than static compression with cooling [19]. The gate control theory of pain explains how non-painful sensory inputs could inhibit pain sensations from being transmitted to the brain [16]. This could explain how compression, especially dynamic compression, works to improve pain management and hypothermia tolerance.

Compression, often used in combination with cryotherapy for care of acute injuries appears to improve tolerability, magnitude and depth of hypothermia. Several studies have repeatedly and meticulously documented that cryocompression results in significantly higher magnitude of surface and even intramuscular temperatures than cryotherapy alone [17, 27]. The enhanced cooling effect with addition of compression may be attributed to the fact that it improves the contact between tissue and cooling interface and also the insulating effect which the layer of compression adds.

We also observed that the efficacy of cryocompression in preservation of peroneal motor nerve function was better than cryotherapy and control (Fig. 3 and Tables 2–3). However, sensory nerve function changes were not found to be different across the three groups [1, 20, 21]. It is important to note, however, that the NCS findings were exploratory in nature and not intended as a primary objective of the study. Moreover, NCS may not be the most reliable method of measuring CIPN [18]. We postulate that the enhanced cooling is a major factor for the vasoconstriction-driven neuroprotection. However, another notable attribute is that compression itself enhances vasoconstriction. Literature reports that compression greater than 30 to 40 mmHg reduces blood flow [17]; however, compression alone would not be able to produce the sufficient vasoconstrictive effect as offered by cryocompression.

Another crucial factor influencing the therapeutic efficacy of hypothermia for chemo-induced toxicities is the therapy protocol. Studies of scalp cooling have shown that initiating cooling 30 to 45 min prior to the administration of chemotherapy is crucial for vasoconstriction-induced neuroprotection [13]. Our temperature data show that the first 60 min is the time period where the maximal cooling occurs after which the tissue attains a thermal stability and hence it is important to attain this state before onset of chemotherapy (Fig. 2) [26]. Moreover, our results concur with that reported by Merrick et al., (1993) in that cryocompression attains a lower temperature faster than cryotherapy (cooling without compression) indicating that compression increases the rate of cooling [17]. Hence, achieving sufficient vasoconstriction before the onset of chemotherapy is a key to enhancing the therapeutic efficacy in alleviating CIPN. As indicated in Fig. 1, our study initiates hypothermia as a pre-cooling phase before the actual golden period, i.e., during chemotherapy, and followed by a post-cooling phase to lock in the vasoconstrictive effect without causing a rewarming effect.

Several studies have employed frozen gloves as a mode of cryotherapy in preventing CIPN [4, 8, 9, 14, 23, 29, 31, 32]. These studies have demonstrated frozen gloves are able to deliver considerable regional hypothermia, however, this effect is not sustained or stable thereby potentially reducing the efficacy of vasoconstriction. Due to the need for change of the frozen gloves every 45–60 min, there is a lack of thermal homeostasis. This process is not only cumbersome but also results in occurrences of intolerance and frostbite [10].

Our study serves as a pilot to demonstrate that cryocompression is a safe and tolerable therapeutic strategy for alleviating CIPN in patients with cancer undergoing taxane treatment. Limitations of the pilot study include a small sample size and the lack of a randomized, prospective control. These findings would need to be validated in a larger prospective study, preferably with randomization. Similar to other supportive care measures such as scalp cooling to reduce CIA, cryocompression may serve to improve the quality of life in cancer patients during and after treatment and also support the delivery of optimal chemotherapy by preventing a dose delay or reduction.

## Electronic supplementary material

ESM 1 (DOCX 2380 kb)

## Abbreviations

CIA: Chemotherapy-induced alopecia
CIPN: Chemotherapy-induced peripheral neuropathy
cMAP: Compound muscle action potentials
CTCAE: Common terminology criteria for adverse events
ECOG: Eastern Cooperative Oncology Group performance status
NCS: Nerve Conduction Study
NCS_pre_: NCS before chemotherapy
NCS_post_: NCS at end of chemotherapy
NCS_3m_: NCS at 3 months after chemotherapy
S.D.: Standard deviation
SAS: Shivering Assessment Scale
SNAP: Sensory nerve action potentials
STS: Subjective Tolerance Scale
TNS: Total Neuropathy Score
VAS: Visual Analogue Scale

## References

[1] Augusto C, Pietro M, Cinzia M, Sergio C, Sara C, Luca G, Scaioli V (2008). Peripheral neuropathy due to paclitaxel: study of the temporal relationships between the therapeutic schedule and the clinical quantitative score (QST) and comparison with neurophysiological findings. J Neurooncol.

[2] Bandla A, Sundar R, Liao LD, Sze Hui Tan S, Lee SC, Thakor NV, Wilder-Smith EP (2016). Hypothermia for preventing chemotherapy-induced neuropathy - a pilot study on safety and tolerability in healthy controls. Acta Oncol.

[3] Barber FA (2000). A comparison of crushed ice and continuous flow cold therapy. Am J Knee Surg.

[4] Beijers A, Mols F, Ophorst J, Pijs J, de Vos-Geelen J, Jacobs E, van de Poll-Franse L, Vreugdenhil G (2017) 1549PD Multicenter randomized controlled trial to evaluate the efficacy of frozen gloves for the prevention of chemotherapy-induced peripheral neuropathy. Ann Oncol 28 10.1016/j.annonc.2019.09.006 31912787

[5] Block JE (2010). Cold and compression in the management of musculoskeletal injuries and orthopedic operative procedures: a narrative review. Open Access J Sports Med.

[6] Cavaletti G, Frigeni B, Lanzani F, Piatti M, Rota S, Briani C, Zara G, Plasmati R, Pastorelli F, Caraceni A, Pace A, Manicone M, Lissoni A, Colombo N, Bianchi G, Zanna C (2007). The total neuropathy score as an assessment tool for grading the course of chemotherapy-induced peripheral neurotoxicity: comparison with the national cancer institute-common toxicity scale. J Peripher Nerv Syst.

[7] Grevelman EG, Breed WP (2005). Prevention of chemotherapy-induced hair loss by scalp cooling. Ann Oncol.

[8] Griffiths C, Kwon N, Beaumont JL, Paice JA (2018). Cold therapy to prevent paclitaxel-induced peripheral neuropathy. Support Care Cancer.

[9] Hanai A, Ishiguro H, Sozu T, Tsuda M, Yano I, Nakagawa T, Imai S, Hamabe Y, Toi M, Arai H, Tsuboyama T (2018). Effects of cryotherapy on objective and subjective symptoms of paclitaxel-induced neuropathy: prospective self-controlled trial. J Natl Cancer Inst.

[10] inc. St (2017) Medical device recall notice: hypothermia caps, mittens and slippers

[11] Kadakia KC, Rozell SA, Butala AA, Loprinzi CL (2014). Supportive cryotherapy: a review from head to toe. J Pain Symptom Manage.

[12] Karandreas N, Papatheodorou A, Triantaphilos I, Mavridis M, Lygidakis C (1995). Sensory nerve conduction studies of the less frequently examined nerves. Electromyogr Clin Neurophysiol.

[13] Komen MM, Smorenburg CH, van den Hurk CJ, Nortier JW (2013). Factors influencing the effectiveness of scalp cooling in the prevention of chemotherapy-induced alopecia. Oncologist.

[14] Loprinzi CL (2017) Prevention and treatment of chemotherapy-induced peripheral neuropathy UpToDate Retrieved from http://www.uptodate.com/home/indexhtml

[15] Massey CS (2004). A multicentre study to determine the efficacy and patient acceptability of the Paxman Scalp Cooler to prevent hair loss in patients receiving chemotherapy. Eur J Oncol Nurs.

[16] Melzack R, Wall PD (1965) Pain mechanisms: a new theory Science. 150:971–979 10.1126/science.150.3699.971 5320816

[17] Merrick MA, Knight KL, Ingersoll CD, Potteiger JA (1993). The effects of ice and compression wraps on intramuscular temperatures at various depths. J Athl Train.

[18] Molassiotis A, Cheng HL, Lopez V, Au JS, Chan A, Bandla A, Leung K, Li Y, Wong K, Suen LK (2019). Are we mis-estimating chemotherapy-induced peripheral neuropathy? Analysis of assessment methodologies from a prospective, multinational, longitudinal cohort study of patients receiving neurotoxic chemotherapy. BMC Cancer.

[19] Murgier J, Cassard X (2014). Cryotherapy with dynamic intermittent compression for analgesia after anterior cruciate ligament reconstruction. Preliminary study Orthop Traumatol Surg Res.

[20] Openshaw H, Beamon K, Synold TW, Longmate J, Slatkin NE, Doroshow JH, Forman S, Margolin K, Morgan R, Shibata S, Somlo G (2004). Neurophysiological study of peripheral neuropathy after high-dose Paclitaxel: lack of neuroprotective effect of amifostine. Clin Cancer Res.

[21] Pachman DR, Qin R, Seisler D, Smith EM, Kaggal S, Novotny P, Ruddy KJ, Lafky JM, Ta LE, Beutler AS, Wagner-Johnston ND, Grothey A, Dougherty PM, Cavaletti G, Loprinzi CL, Staff NP (2016). Comparison of oxaliplatin and paclitaxel-induced neuropathy (Alliance A151505). Support Care Cancer.

[22] Ping Ng KW, Ong JJ, Nyein Nyein TD, Liang S, Chan YC, Lee KO, Wilder-Smith EP (2013). EMLA-induced skin wrinkling for the detection of diabetic neuropathy. Front Neurol.

[23] Sato J, Mori M, Nihei S, Kumagai M, Takeuchi S, Kashiwaba M, Kudo K (2016). The effectiveness of regional cooling for paclitaxel-induced peripheral neuropathy. J Pharm Health Care Sci.

[24] Seretny M, Currie GL, Sena ES, Ramnarine S, Grant R, MacLeod MR, Colvin LA, Fallon M (2014). Incidence, prevalence, and predictors of chemotherapy-induced peripheral neuropathy: a systematic review and meta-analysis. Pain.

[25] Sundar R, Bandla A, Tan SS, Liao LD, Kumarakulasinghe NB, Jeyasekharan AD, Ow SG, Ho J, Tan DS, Lim JS, Vijayan J, Therimadasamy AK, Hairom Z, Ang E, Ang S, Thakor NV, Lee SC, Wilder-Smith EP (2016). Limb hypothermia for preventing paclitaxel-induced peripheral neuropathy in breast cancer patients: a pilot study. Front Oncol.

[26] Tikuisis P (2003). Heat balance precedes stabilization of body temperatures during cold water immersion. J Appl Physiol.

[27] Tomchuk D, Rubley MD, Holcomb WR, Guadagnoli M, Tarno JM (2010). The magnitude of tissue cooling during cryotherapy with varied types of compression. J Athl Train.

[28] Wampler MA, Miaskowski C, Hamel K, Byl N, Rugo H, Topp KS (2006). The modified total neuropathy score: a clinically feasible and valid measure of taxane-induced peripheral neuropathy in women with breast cancer. J Support Oncol.

[29] Wilkinson M, Cocilovo C, Vargas HI, Cohen RA, Bruce S, Edmiston KH, Franco CY, Agra MG, Bayer S, Khan A (2016) Reduction of paclitaxel neuropathy with cryotherapy

[30] Windebank AJ, Grisold W (2008). Chemotherapy-induced neuropathy. J Peripher Nerv Syst.

[31] Yamashita T, Hattori M, Nakada T, Hayashi T, Kamei K, Tatsuya T, Nagao Y, Mase T, Wada M, Mizuno T (2019) Abstract P4-11-02: Subjective and objective assessment of efficacy of frozen gloves and socks to prevent nab-paclitaxel-induced peripheral neuropathy in patients with breast cancer. 79:P4-11-02

[32] Younus J, Kligman L, Jawaid D (2016). The impact of cold therapy on the incidence and severity of paclitaxel induced peripheral neuropathy: a pilot study. J Solid Tumors.

