# Supplementary material for: Safety and tolerability of cryocompression as a method of enhanced limb hypothermia to reduce taxane-induced peripheral neuropathy
Source: Support Care Cancer. 2019 Dec 6;28(8):3691–9. doi: 10.1007/s00520-019-05177-2 (PMC7316694; doi:10.1007/s00520-019-05177-2)
Supplement: Supplementary file 1 — (DOCX 2380 kb) [file 520_2019_5177_MOESM1_ESM.docx]

**Safety and Tolerability of Cryocompression as a Method of Enhanced Limb Hypothermia to Reduce Taxane-Induced Peripheral Neuropathy**

Aishwarya Bandla^1^, Stacey Tan^1^, Nesaretnam Barr Kumarakulasinghe^2^, Yiqing Huang^2^, Sally Ang^2^, Gayathiri Magarajah^1^_,_ Zarinah Hairom^3^, Joline Si Jing Lim^2^, Alvin Wong^2^, Gloria Chan^2^, Natalie Ngoi^2^, Emily Ang^3^, Yee Mei Lee^3^, Amanda Chan^4^, Soo-Chin Lee^2,5^, Nitish Thakor^1,6^, Einar Wilder-Smith^1,4,7*^, Raghav Sundar^1,2,8*^

^1^The N.1 Institute for Health, National University of Singapore, Singapore, Singapore.

^2^Department of Haematology-Oncology, National University Health System, Singapore, Singapore.

^3^National University Cancer Institute, National University Health System, Singapore, Singapore.

^4^Department of Medicine, National University Health System, Singapore, Singapore.

^5^Cancer Science Institute of Singapore, National University of Singapore, Singapore.

^6^Department of Biomedical Engineering, Johns Hopkins University, Baltimore, U.S.A.

^7^Neurology, Kantonsspital Lucerne, Switzerland.

^8^ Yong Loo Lin School of Medicine, National University of Singapore, Singapore.

*Correspondence should be addressed to the following:

Prof. Einar P. V. Wilder-Smith

Department of Medicine,

National University Health System, Singapore, Singapore.

Tel.: (65) 6772 4380; E-mail: [einar_wilder-smith@nuhs.edu.sg](mailto:einar_wilder-smith@nuhs.edu.sg)

Dr. Raghav Sundar

Department of Haematology-Oncology,

National University Health System, Singapore, Singapore.

Tel.: (65) 6772 7580; E-mail: [raghav_sundar@nuhs.edu.sg](mailto:raghav_sundar@nuhs.edu.sg)

Supplementary Material

**Supplementary Tables**

**Table S1.** Adverse events.

| **Adverse events** | **Number of cases (N = 13)** | |
| --- | --- | --- |
|  | **All grades** | **Grades 3-4** |
| Sensory neuropathy | 7 | 0 |
| Grade 0 | 6 | - |
| Grade 1 | 7 | - |
| Haematological |  |  |
| Neutropenia | 2 | 2 |
| Anaemia | 0 | 0 |
| Renal impairment |  |  |
| Creatinine increased | 0 | 0 |
| Hypokalaemia | 0 | 0 |
| Dysuria | 0 | 0 |
| Nails |  |  |
| Nail discoloration | 0 | 0 |
| Nail infection | 0 | 0 |
| General skin toxicity  (not over region of cooling) |  |  |
| Zoster | 0 | 0 |
| Excoriations | 0 | 0 |
| Rashes | 2 | 0 |
| Erythema | 0 | 0 |
| Dermatitis | 8 | 0 |
| Skin reaction | 0 | 0 |
| Arthralgia/myalgia | 3 | 0 |
| Head and brain |  |  |
| Headache | 2 | 1 |
| Fatigue | 9 | 0 |
| Dizziness | 1 | 0 |
| Insomnia | 1 | 0 |
| Nose, mouth and throat |  |  |
| Gingivitis | 0 | 0 |
| Toothache | 2 | 0 |
| Oral mucositis | 1 | 0 |
| Dysgeusia | 1 | 0 |
| Pharyngitis | 1 | 0 |
| Respiratory |  |  |
| Upper respiratory tract infection | 3 | 0 |
| Dyspnoea | 1 | 0 |
| Cough | 2 | 0 |
| Gastrointestinal |  |  |
| Diarrhoea | 2 | 0 |
| Constipation | 0 | 0 |
| Vomiting | 1 | 0 |
| Dyspepsia | 0 | 0 |
| Reflux | 0 | 0 |

**Supplementary Figures**


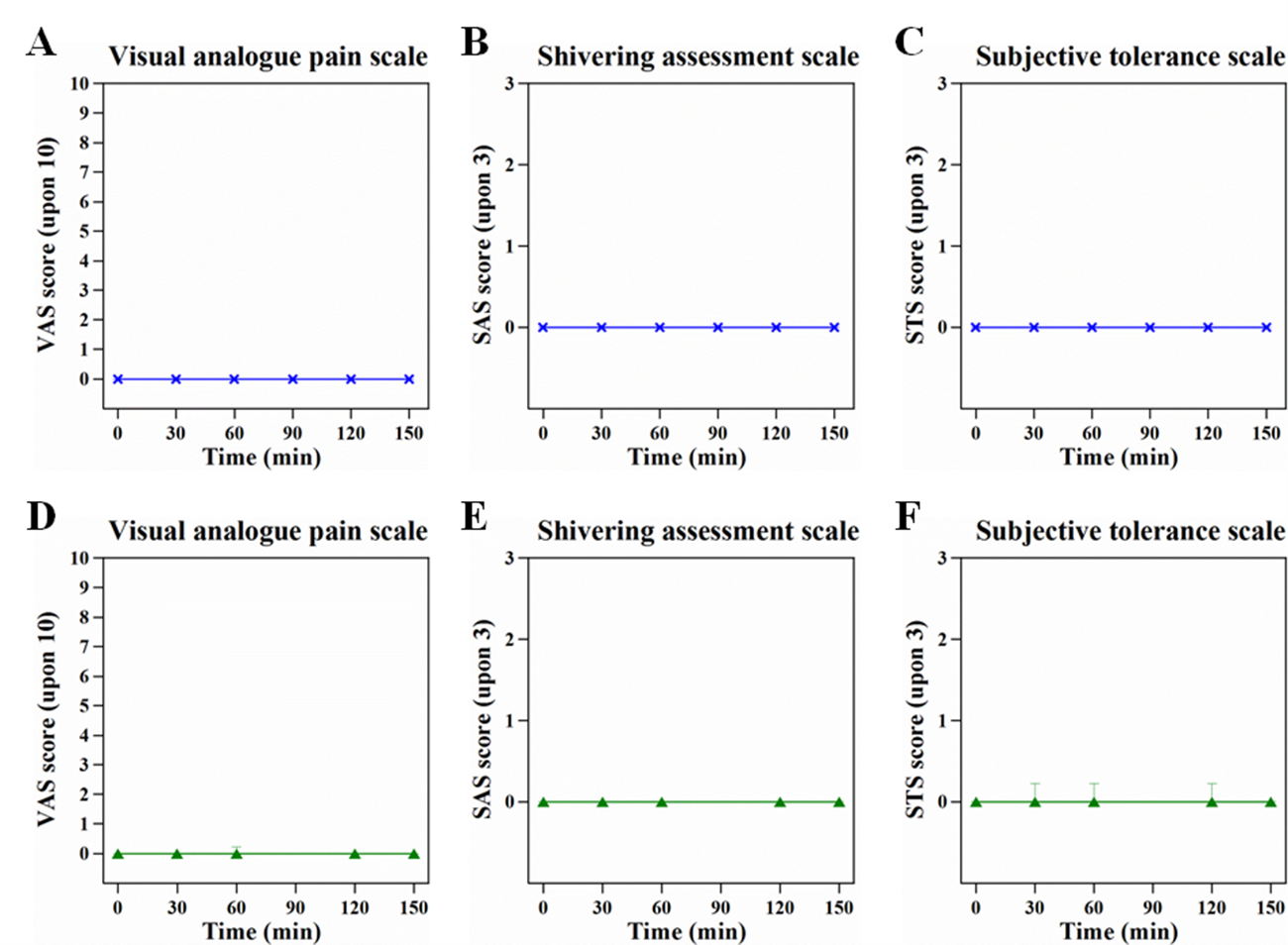


**Fig. S1** Trend of tolerability scores reported throughout the duration of limb hypothermia. Visual analogue scale, shivering assessment scale and subjective tolerance scale are depicted. Sub figures (a-c) depict the scores for subjects who underwent cryocompression at 16°C and (d-f) indicate tolerance scores of patients who underwent continuous-flow cooling at 22°C and without compression.


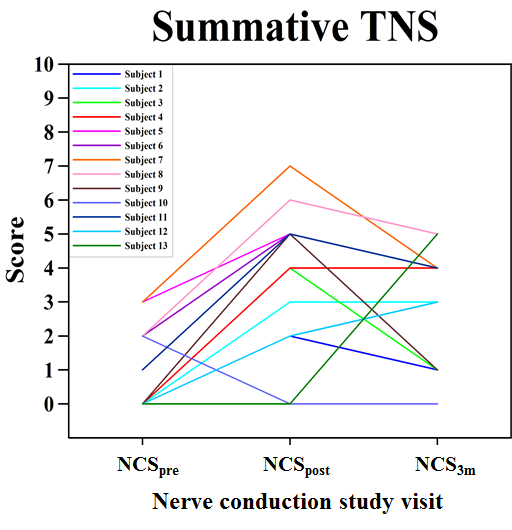


**Fig. S2** Summative TNS score trends of each patient over the three NCS visits.


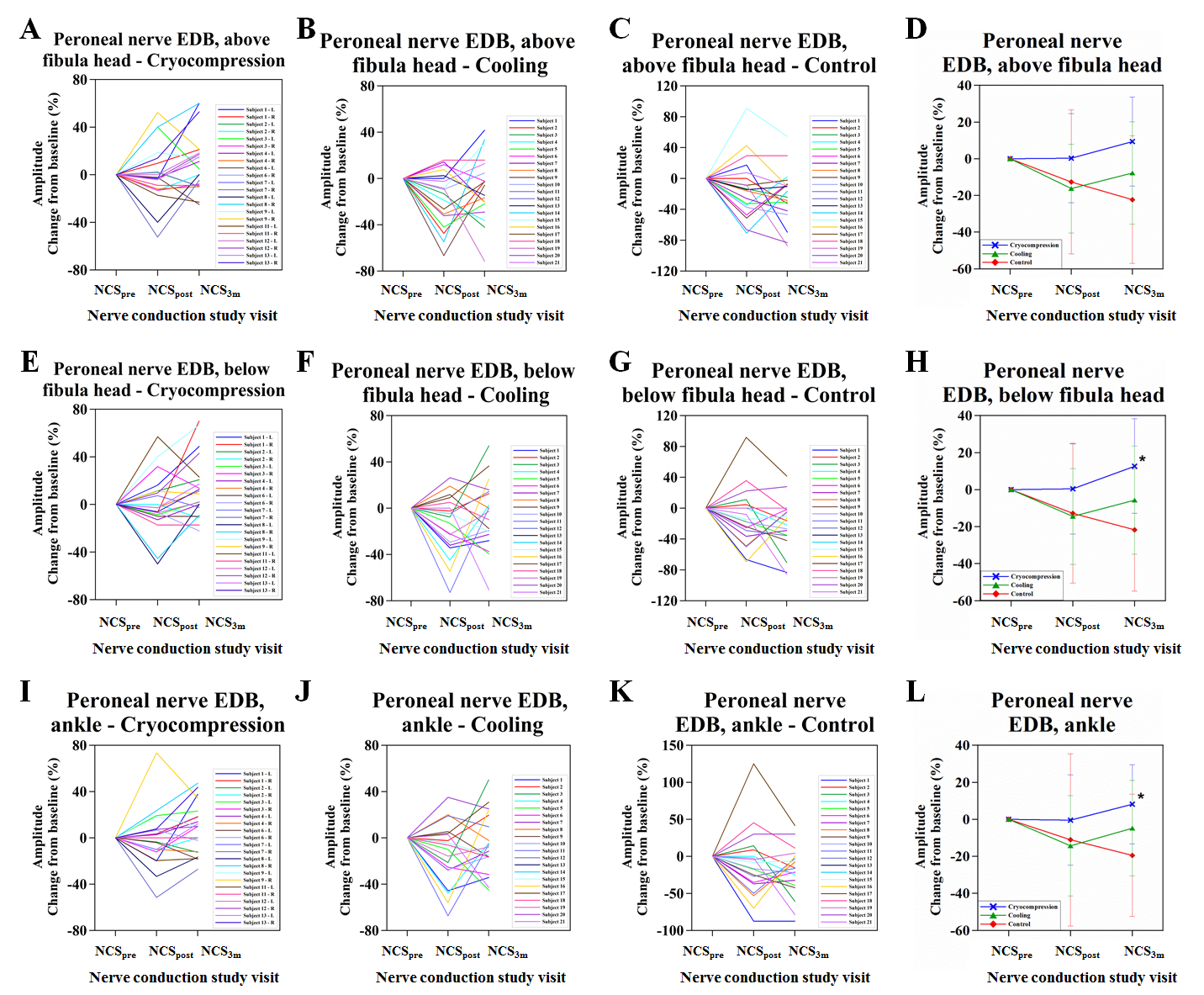


**Fig. S3** Spaghetti plots of changes in nerve conduction amplitudes in the motor nerves illustrated in Figure 3, at three time points – before, end of chemotherapy and after three months. Changes with cryocompression (a, e, i) and cooling (b, f, j) techniques and without limb hypothermia (c, g, k). Overall, the changes (d, h, l) indicate that cryocompression (blue) better preserves motor nerve conduction amplitudes compared to continuous-flow cooling (green). The non-cooled limb was considered as control (red), continued to deteriorate. ^**^ indicates *p* <0.01 and ^***^ indicates *p*<0.001.
